# Effects of Foliar Protector Application and Shading Treatments on the Physiology and Development of Common Bean (*Phaseolus vulgaris* L.)

**DOI:** 10.3390/plants13141968

**Published:** 2024-07-18

**Authors:** Cleiton Sousa, Kenia Trindade, Ederlon Moline, Luiz Enrick Rocha De Lima, Sara Bernardo, Hyrandir Cabral de Melo

**Affiliations:** 1 Instituto Federal Goiano, Campus Ceres, Ceres 76300-000, GO, Brazil; luiz.enrick@estudante.ifgoiano.edu.br; 2 Programa de Pós Graduação em Irrigação no Cerrado, Instituto Federal Goiano, Campus Ceres, Ceres 76300-000, GO, Brazil; kenialorrany@hotmail.com; 3 Santa Clara Agrociência Industrial, Av. Cel. Fernando Ferreira Leite, 305, Ribeirão Preto 14026-010, SP, Brazil; ederlon.moline@santaclaraagro.com.br; 4 Centre for the Research and Technology of Agro-Environmental and Biological Sciences (CITAB), University of Trás-os-Montes and Alto Douro, 5000-801 Vila Real, Portugal; sbernardo@utad.pt; 5 Instituto de Ciências Biológicas, Universidade Federal de Goiás, Goiânia 74690-900, GO, Brazil; hyrandir@ufg.br

**Keywords:** *Phaseolus vulgaris*, solar radiation, photoprotection, gas exchange and productivity

## Abstract

High solar radiation, combined with high temperature, causes losses in plant production. The application of foliar protector in plants is associated with improvements in photosynthesis, reduction in leaf temperature and, consequently, improved productivity. Two experiments were conducted. The first aimed to assess the efficacy of foliar protector versus artificial shading in mitigating the negative impacts of excessive radiation and temperature on the physiology, growth, and yield of common bean plants. The second experiment focused on comparing the timing in cycle plants (phenological phases) of foliar protector application in two different bean cultivars (BRS Fc 104 and BRS MG Realce) under field conditions. Artificial shading provided better results for photosynthesis, transpiration, growth and production compared to the application of foliar protector. In the field conditions experiment, the application timing of the foliar protector at different phenological phases did not increase productivity in the cultivars. The application of foliar protector under the conditions studied was not effective in mitigating the negative impacts of high solar radiation and temperature on common bean cultivation. However, it is opportune to evaluate the application of foliar protector in bean plants grown under conditions with water deficit, high solar radiation and high temperature.

## 1. Introduction

Solar radiation is essential for plant production. However, in tropical regions, the incoming photosynthetic photon flux density (PPFD) reaches values above 2000 µmol m^−2^ s^−1^ during the dry season (May–September), causing thermal and light stress mainly in C3 plants, such as *Phaseolus vulgaris* L. [1]. Common bean is a well-known legume crop in Brazil, and is one of the pillars of the human diet, cultivated in the different edaphoclimatic conditions of the country, which shows the broad adaptability and morphophysiological diversity of the cultivated genotypes. In the Brazilian Cerrado, it is recommended to cultivate between September and November, January and March or May and July, the latter making it possible to obtain higher yields from the crop [2]. Among the damages caused by high temperatures, the closing of stomata, reduction in carbon assimilation, and the increase in photorespiration stand out, which can compromise more than 40% of net photosynthesis [3]. Likewise, excessive radiation can also cause photoinhibition, damage to the photosynthetic apparatus and an increase in leaf temperature, resulting in lower accumulation of biomass and photoassimilates, as demonstrated in a recent study undertaken in twelve bean genotypes exposed to heat stress (26–37 °C) after flowering [4]. Under high light conditions, the maximum quantum yield of photosystem II (Fv/Fm) reduces along with a decrease in the photochemical rate, and an increase in the rate for non-photochemical dissipation of excess excitation energy [5]. Light saturation responses, photosynthetic capacity, and sensitivity to diurnal variations in air temperature, leaf temperature, and vapor pressure deficit among different common bean genotypes were compared [6]. High temperature also influences photoinhibition by modulating CO_2_ concentration, and its combination with other environmental stress factors (e.g., high light and drought) can promote distinct effects. In order to take advantage of the genetic potential of plants and obtain higher yields in the context of climate change, environmental control prevails in intensive cultivations, as well as the use of other planting and management technologies in extensive cultivations [7].

The application of foliar protectors is associated with the reduction in damage caused by excessive solar radiation and high temperature [8], increasing the yield and quality of several crops [9]. In the market, products based on calcium and potassium silicates, calcium and magnesium hydroxide, calcium carbonate, and aluminum silicate predominate. Once applied at the leaf level, a particle film is formed on the surface, increasing the reflectance, water efficiency, and reducing transpiration, absorbance of solar radiation and leaf temperature [10].

These principles motivate the application of foliar protectors in different crops, even without fully validating the benefits indicated for some species grown in different type-climate regions. The spraying of a product based on aluminum silicate (kaolin) increased carbon assimilation by the grapefruit (*Citrus*) leaf under high radiation and temperature stress [11]. In strawberry seedlings, it improved photosynthesis and the initial and total weight of marketable fruits [9]. Kaolin application benefited leaf cooling, slightly reduced photosynthesis rates and water loss in vines grown without water stress [12]. Under conditions of water deficit and subsequent re-irrigation, they observed the grapevines’ ability to protect foliar function by avoiding photoinhibition and maintaining effective evaporative cooling even at the peak of water stress. The application of kaolin reduced thermal stress at different times of the day and provided a reduction of 8.5 to 13.8% in leaf temperature [13]. In beans (cv Dragone), kaolin application from the beginning of flowering until harvest reduced stomatal conductance and transpiration and increased water use efficiency by 20% [14]. These studies indicate that kaolin photoprotective effects and positive impacts on yield are more effective under high temperature and/or heat stress. This probably occurs with the increase in reflective power, reducing the leaf temperature, and consequently, the thermal stress, but also reducing the light available for photosynthesis, allowing for the compensation of the benefits of the lower temperature with high incident radiation [15]. The application of kaolin caused a decrease in abscisic acid and an increase in auxins concentration, associated with the improvement in the physiological performance of grapevine under heat stress [13]. More recently, Bernardo et al. (2021) [16] showed that kaolin application could improve the plasticity of grapevine stress responses to severe environmental conditions by fine-tuning the xanthophyll cycle. The use of nanoparticle technology in agriculture has been increasingly explored in recent years as an alternative strategy to reduce fertilizer inputs and enhance yield and quality in a changing climate. Most studies on bean plants focused on the foliar application of micronutrients as nanofertilizers, upgrading the vegetative growth, flower number/plant, photosynthetic pigments and yield, [17,18] as well as the use of silver nanoparticles.

Moreover, the application of calcium nanoparticles has also been found to improve drought stress tolerance in *Brassica napus* seedlings. However, there is still a lack of consistent evidence regarding the effects of nanoparticle application in avoiding sunburn and high-light damages in common bean production benefiting the physiological performance of the plants. In this sense, this study focused on comparing two different strategies, a commercial foliar protector spray and artificial shading at two levels, applied in common beans to avoid high-light and temperature damage by monitoring photosynthesis, transpiration and growth traits. In addition, to evaluate the efficiency of the foliar protector under field conditions, a second experiment was performed in a cultivation field to apply a protector at various phenological stages in two different varieties.

## 2. Results

The FP was applied to mitigate the negative impacts of excessive radiation and temperature on the physiology, growth, and yield of the plants common bean. Two experiments were conducted to assess the effectiveness of foliar application of FP on common bean plants. One experiment was carried out in a controlled environment (pot experiment), while the other was conducted under field conditions.

**Figure 1:**
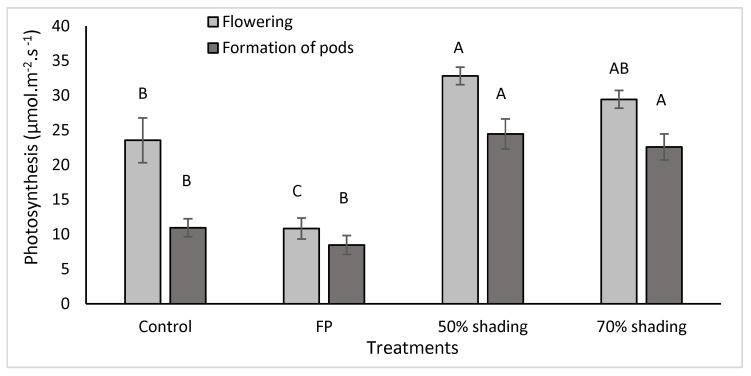
Net photosynthesis (µmol m^−2^ s^−1^) at 31 and 48 DAE of common bean (BRS Fc104) control and FP-treated plants. Different letters denote significant differences between treatments (control, foliar protector—FP, and artificial shading) within each developmental stage (31 DAE and 48 DAE) by Tukey’s test at a 5% probability of error.

**Figure 2:**
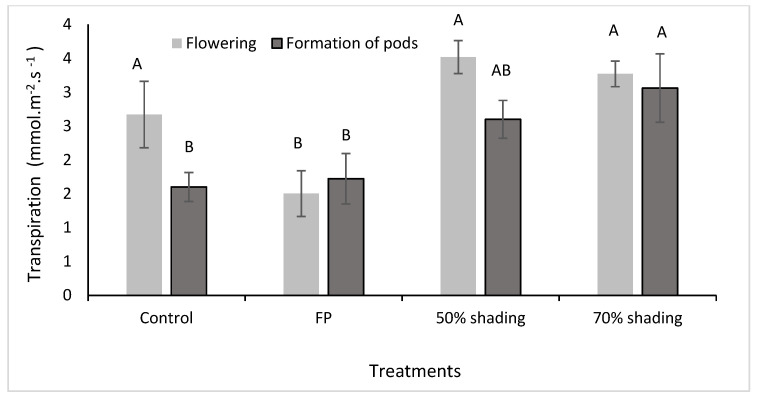
Transpiration rates in two phenological phases (31 DAE and 48 DAE) of common bean (BRS Fc104) with or without foliar protector application. Within each phenological stage, means followed by the same letter do not differ statistically by Tukey’s test at a 5% probability of error.

In the pot experiment, foliar protector (FP) application and artificial shading influenced net photosynthesis and transpiration in common beans. The artificial shading provided greater photosynthesis and transpiration contrasting with the observed effects of FP application (Figure 1 and Figure 2).

Five days after the first FP application, in phase R6 (beginning of flowering), the FP application reduced photosynthesis by 53.9% compared to the control, while a 50% shading treatment increased photosynthesis by 39.38%. In phase R7 (beginning of pod formation), FP treatment showed no significant effect on photosynthesis compared to control, while shading with 50% increased photosynthesis by 123.1% (Figure 1). In phase R6, FP application reduced transpiration by 43.74% compared to control plants and no significant effect was found at beginning of pods formation. The active photosynthetic radiation of readings on plants in full sun was 2283.27 μmol·m^−2^·s^−1^ in phase R6 and 1715.82 μmol·m^−2^·s^−1^ in phase R7.

With artificial shading, the transpiration rates at flowering increased 31.7% with shading of 50%, while in phase R7 (48 DAE), shading with 70% increased 91.15% in transpiration compared to control (Figure 2). There was no significant difference between shading treatments at both phases, but transpiration levels were generally higher than the ones of FP treatments.

**Figure 3:**
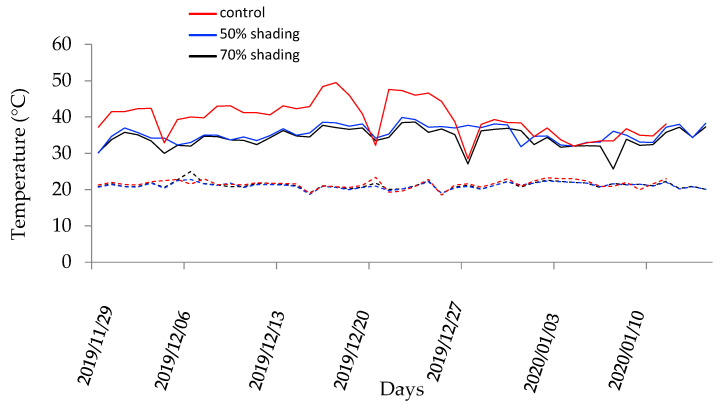
Maximum and minimum air temperatures (°C) in environments in common bean cultivation in Ceres-GO, from November 2019 to January 2020. (Solids lines indicate maximum temperature and dash line minimum temperature).

The maximum temperature in environments protected with commercial screens with 50 and 70% shading was similar and lower than in full sun, respectively (Figure 3).

Environments with 50 and 70% shading showed little variation in maximum and minimum temperatures. The minimum temperature showed low variation during cultivation and between cultivation environments. For the maximum temperature, a greater amplitude was observed during the experiment and lower values in environments with 50 and 70% shading in relation to full sun (Figure 3). In full sun, the maximum temperature exceeded 45 °C, which could compromise the physiology, growth and productivity of plants. In environments with 50 and 70% shading, the maximum temperatures during cultivation were similar, and could provide the same responses for photosynthesis and transpiration.

Growth and yield parameters are shown in Table 1. The shading of 50 and 70% provided greater plant height (PH), distance between the second and the third node (DBSTN), distance between the third and the fourth node (DBTFN), and distance between the fourth and the fifth node (DBFFN) compared to control and FP application. The distance between the first and the second node (DBFSN), first pod insertion height (FPIH) and stem diameter (SD) showed no difference between the cultivation environments (Table 1).

The shading treatments increased growth and production variables. Shading conditions of 50 or 70% provided more pods per plant (NPP) than FP application. The highest values for the number of grains per plant (NGP) and grain mass per plant (GMP) were obtained with 70% shading. Although shading provided higher plant dry mass (PDM) compared to FP application, no significant difference was observed regarding control. The application of FP resulted in a 10.4% reduction in stem diameter compared to the control (Table 1).

The increase in the number of branches did not correspond to an increase in the number of pods per plant and productivity (Table 2), indicating the plant’s compensatory capacity. The number of pods per plant and the number of grains per plant correlated positively with the grain mass per plant (0.88 and 0.99, respectively), as well as the number of pods per plant with the number of grains per plant (0.92). The growth variables did not correlate positively with the accumulation of dry mass in plants and grain production (Table 2).

In the field experiment, the production of cultivar BRS Fc 104 showed the highest number of branches, distance between nodes, number of pods per plant, number of grains per plant and highest productivity (Table 3), showing significant genotypic differences between the cultivars. The increase in the number of shoots provides a greater quantity of plant tissues that can differentiate into reproductive structures, resulting in a greater number of flowers, pods, number of grains per plant and greater productivity (Table 3).

FP application did not influence branching in the BRS MG Realce cultivar, while in BRS Fc 104, a reduction in the number of branches was observed with application in flowering, pod formation, in the combination of pod formation + grain filling and vegetative + flowering + phases. Application of FP at pod formation + grain filling (Table 4), however, reduced the number of shoots and did not correlate with productivity losses (Table 5).

In cultivar BRS Fc 104, FP application in the grain filling phase increased productivity by 6.5%, and when applied in the pod formation phase (R6), it reduced productivity by 19.15%. In the BRS MG Realce cultivar, application in the pod formation phase (R6) increased 10.36%, while application in flowering reduced productivity by 23.8% (Table 5). The responses obtained in the two cultivars of the *Phaseolus vulgaris* highlight the need to establish the ideal time for applying the foliar protector, as, depending on the time of application, it can favor or harm productivity.

## 3. Discussion

The application of foliar protectors generally forms a film on the surface of leaves or fruits, reducing water loss and CO_2_ entry. In hypostomatic species, generally, its use does not obstruct the stomata, allowing for a greater reflection of radiation and thermoregulation without compromising transpiration. In amphistomatic species, however, it can obstruct the stomata and reduce transpiration and photosynthesis [19]. Common bean is an amphistomatic plant, and probably, the stomatal conductance of the adaxial part of the leaves was limited with FP application, and consequently, the entrance of CO_2_ for the Calvin Cycle, resulting in lower photosynthetic rates [10].

Water evaporation in the full-sun environment was higher than in shaded environments and, consequently, greater transpiration in plants is expected; however, this did not occur (Figure 2). The greater evaporation of water in the full-sun environment probably occurred due to the high solar radiation and higher temperature, resulting in changes in stomatal conductance and a lower transpiration rate to avoid excessive water loss.

Cultivar BRS Fc 104 has a highly early cycle, taking 60 to 65 days between emergence and physiological maturation. At 48 DAE net, photosynthesis was lower than that at 31 DAE. The greater demand for photoassimilates in plants predominates in the formation of reproductive structures, with a peak in photosynthesis, followed by a reduction in photosynthetic activity. At 48 DAE, the beginning of changes in the color of the pods and senescence of the older leaves were already observed, and the formation of the grains was probably already finishing. With leaf aging, there is a reduction in metabolic activity and, consequently, a reduction in the photosynthetic rate [20], resulting in differences between the flowering and formation the pods phases.

This study demonstrates that shading at 50% favored photosynthesis at 31 and 48 DAE compared to plants grown in an open field (control) and with FP application (Figure 1). Interestingly, at 31 days after emergence, the application of FP resulted in reduced transpiration rates. However, by 48 days after emergence, shading at 70% led to increased transpiration compared to the control and FP application (Figure 2). These findings suggest that careful manipulation of light can potentially enhance plant growth and productivity with the optimization of conditions for photosynthesis and transpiration.

The photosynthetically active radiation of 1500 µmol m^−2^ s^−1^ caused photoinhibition in *Vicia faba* leaves and, consequently, reduced photosynthetic activity [21]. It was reported that both high and low temperatures increase photoinhibition, manifested by a decrease in Fv/Fm, and that, in each temperature condition, the plant adopts a specific pattern for the recovery of the photosynthetic apparatus after photoinhibition [22]. Therefore, it is highlighted that the climatic conditions in the Cerrado have a great potential to induce photoinhibition in beans, whether due to high solar radiation and temperature, requiring studies to establish management strategies aimed at mitigating such effects on agricultural crops.

In general, the results indicate that common bean did not achieve optimal photosynthesis and transpiration rates when grown under full-sun conditions (control), likely due to excessive solar radiation in the environments (Figure 1, Figure 2 and Figure 3). During pod formation, the time of greatest demand for photoassimilates, there is a greater impact of cultivation in full sun and FP application on photosynthesis and transpiration.

Common bean reaches saturation with photosynthetically active radiation between 900 and 1000 μmol m^−2^s^−1^ [23]. High light intensity can increase photoinhibition, ROS production and cause irreversible damage to the photosynthetic apparatus. To minimize damage, there is an increase in the production of photoprotective pigments and movement of chloroplasts in the leaves, altering the photosynthetic capacity. In addition, chlorophyll and carotenoid contents may increase with shading, allowing for a better use of solar radiation [24].

Furthermore, the application of FP did not provide equally effective results when compared to shading treatments. These results suggest that the use of FP can compromise gas exchange, growth, and productivity in plants and, therefore, its recommendation should be limited to stressful conditions and in extensive crops and/or with a low technological level due to its ability to reduce transpiration and consequently water loss.

*Jatropha curcas* L. plants grown in different light conditions, in an open field, had a greater number of leaves, stem thickness, higher net assimilation rates and carboxylation efficiency in full sun [25]. These results were obtained in the municipality of Capão do Leão, RS. This demonstrates the need to define strategies to obtain better growth and physiological responses for each species and in different edaphoclimatic conditions, considering that solar radiation ranges more than 2400 μmol m^−2^s^−1^ at the experiment location.

Transpiration is directly associated with stomata opening and indirectly with leaf temperature. Water evaporation from the leaf increases at higher temperatures. The maximum temperature in the open field, for most of the experimental period, was higher than the maximum temperature in the shaded environments (Figure 3).

At 31 DAE, the maximum temperature in open field was higher than in the other environments, approaching 50 °C (Figure 3), and transpiration was lower than in shaded environments (Figure 2). At 48 DAE, there was a small fluctuation in the maximum temperature in the three environments, below 40 °C; however, transpiration in the environment with full sun and 50% shading was lower than in the environment with 70% shading. The minimum temperature did not show significant variation between environments (Figure 3).

Due to the high light incidence in plants grown under full sun, there was a disproportion of photons available for photosystem I and photosystem II [26], affecting their subunits and reducing the expression of the Rubisco minor subunit [27], thus altering the photosynthetic performance. With the photosynthetic capacity of plants impaired, some of the absorbed energy was dissipated through alternative pathways, such as heat, highlighting the necessity to refine the mechanism for dissipating absorbed light energy [28]. In addition, we can infer that, as a consequence of this reduction in transpiration, there may have been an increase in leaf temperature, since transpiration, in addition to acting on gas exchange, also acts on leaf cooling, that is, it acts by controlling leaf temperature [29].

At 48 DAE, after the second application of FP, it was observed that the cultivation environments influenced the photosynthesis and transpiration parameters. Under full-sun conditions (control), photosynthesis and transpiration were lower, probably due to stomatal closure, resulting in lower production of photosynthesis [30]. Cultivar BRS Fc 104 presents a good condition for adapting the photosynthetic apparatus to different cultivation environments; in particular, it is mentioned that, in shaded environments, there is a greater accumulation of resources for vegetative organs, affecting productivity, emphasizing that there was an increase of 5% in PSII potential (Fv/Fm) in plants grown at 50% shading [31].

The photosynthesis and transpiration, after the second application, was influenced only by the cultivation environments. Transpiration was lower in environments with full sun (control) and with the application of FP compared to environments with 70% shading. This also demonstrates that shading treatments were more effective than FP in responding to cultivation conditions as they allowed plants to continue to transpire and keep their stomata open, contributing beneficially to photosynthesis (production of photoassimilates).

The productive cycle of the BRS Fc 104 cultivar lasts approximately 75 days from sowing to harvest; however, in all environments, this average period was anticipated. The anticipation of the cycle of cultivar BRS Fc 104 may be associated with the influence of light on the time required for the occurrence of phenophases.

The interaction between FP application and cultivation environments did not influence the growth and production variables of the common bean cultivar BRS FC 104. The cultivation environments, on the other hand, influenced plant height, distance between nodes, number of pods per plant, number of grains per plant, grain mass per plant, and dry mass of plants (Table 1).

Considering that the experiment was conducted in summer, with high temperatures and high solar radiation, the conditions were favorable to a decrease in CO_2_ fixation with stomatal closure in plants in full sun, causing a reduction in C allocation in leaves and growth tissues [32].

The shading favored an increase in photosynthesis and, consequently, the accumulation of dry mass (Table 1), and provided greater plant height, length between the second, third and fourth nodes and accumulation of plant dry mass (Table 1). This accumulation of dry mass indicates a positive carbon balance in the plant since the carbon that was fixed is being directed to the accumulation of biomass [33].

Plant height and distance between nodes are associated with cell division and expansion, which are controlled by the action of plant hormones and photoreceptors. It is possible that low light intensity, as well as long days, increased the response capacity of endogenous gibberellins, inducing an increase in the response capacity of cell tissue and meristematic regions [34], causing elongation between nodes by cell expansion and division [35].

The height of insertion of the first pod was not influenced by treatments, possibly due to the formation of tissues, which gave rise to the pods, at the beginning of plant growth, before the effects of treatments, and the factor genetics predominated in the determination of the response. The insertion height of the first pod directly influenced the number of pods that the plant produced. Plants with the first pod closer to the base of the soil supported a greater number of pods. In soybean cultivation in different shading, it was possible to observe that the higher the shading level, the taller the plants were due to a greater cell elongation developed by the plants, and the shading level that presented the most significant difference was 50% of radiation interception [36].

The distance between the nodes shows the growth and development of the plant, in addition to the natural development of some physiological factors that may change due to the environment to which the plants are subjected. The distance between the second and the third nodes and the distance third and fourth nodes showed similar results, with the best average obtained with 50% and 70% environments.

Sprouting is associated with cytokinin levels, being favored with light. On the other hand, in full-sun conditions, it compromised the number of pods per plant, possibly due to higher temperature, transpiration and lower photosynthesis. Pearson’s correlation showed that shading favored the number of pods in relation to the application of sunscreen and in full sun (control). Regarding dry mass accumulation, positive contributions from plant height and stem diameter stood out.

Plant growth and development is affected by light intensity, quality, period and temperature, including the synthesis and production of hormones such as endogenous gibberellins, inducing an increase in the responsiveness of cell tissue and meristematic regions, an increase in the area foliar, in order to capture more light to combat the low photosynthetic capacity and an increase in secondary metabolism [37]. Similar results have been reported in other species, including *Solanum lycopersicum* L., [38]. Increased shoot length in plants exposed to low light has been associated with controlling the balance of AIA oxidase enzyme activity, in which red pigment activation triggers oxidative enzyme systems that convert the AIA oxidase enzyme cofactors that serve as inhibitors, promoting an increase in endogenous auxins [39].

The number of pods per plant, number of grains per plant and grain mass per plant are correlated variables, with these three variables having the highest averages in the environment with 70% shading. The number of pods per plant had the highest average at 70% shading, with a value of 18.58, followed, respectively, by the means of 50% shading (8.2) and full sun (3.25). It is observed that the lower the incidence of light, the greater the influence on the development of pods. For number of grains per plant, the 70% shading environment obtained 56.58, and only 3.8 in full sun (control) and 3.75 with FP application.

For grain mass per plant, the environment with 70% shading showed an average of 13.58 g. Grain mass is reflected in production, as heavier grains result from greater accumulation of by-products arising from the photosynthetic process. Dry mass is all material resulting from the drying (removal of water) of plants under controlled conditions. For the present study, the best averages obtained occurred in environments from 50% to 70%, with 13.3 g and 13.75 g, respectively. In the environment in full sun (control), the plants grew less, there was less production of sprouts, and consequently less leaf production, which may have led to a lower average (8.08 g) in relation to the other environments.

These results indicated that a greater incidence of shading during cultivation favored parameters related to both physiological performance, growth and productivity in the BRS Fc104 cultivar, indicating that the reduction in light incidence in plants of this cultivar led to a better performance. With this, it is emphasized that intercropping with crops that provide shading can benefit producers’ profitability by allocating growth resources, such as nutrients, moisture and light interception [40], as observed with the promotion of intercropping of beans with corn [41].

FP application was not capable of altering the growth and development in bean plants under the conditions used in this experiment, with only the results arising from the shading condition being evidenced. The respective results provide new perspectives for common bean production, indicating that production in areas with less light incidence implies obtaining greater productivity and physiological performance of cultivars.

In Brazil, there is an expansion of the Integrated Crop, Livestock and Forestry System and the cultivation of corn, soybeans and grass predominates. With the possibility of reducing solar radiation with the forest species, there is evidence of improving conditions and obtaining greater productivity of common bean in ILPF cultivation.

FP application in irrigated common bean did not provide benefits to the crop. The results indicate the need to study FP application in plants grown under water deficit conditions. The water supply with irrigation, either with drip or central pivot, provides favorable conditions for gas exchange, growth and production of common bean. Thus, in cultivation with a low technological level, and unfavorable environmental conditions, FP application could have an effect in mitigating the lack of water and high temperature.

Common bean presents distinct branching between growing conditions. The points of insertion of pods predominate at the base of the plant, around a maximum of 2/3 of the plant. Thus, the leaves of the final third of the canopy of the plants can act as a filter to solar radiation, providing specific environments for the source leaves with greater potential for the production of photoassimilates to meet the demands of the drains, such as in grain filling. In this sense, FP application on plants in conditions of water deficit and with lower leaf density in the canopy can more effectively attenuate the effects of excess radiation.

In field cultivation, differences between cultivars are probably associated with genetic factors. FP application did not increase the production of common bean cultivars. The productivity of cultivar BRS Fc 104 may have promoted a higher number of sprouts, and consequently a greater number of pods and number of grains per pod (Table 2). The greater distance between nodes, associated with a smaller stem diameter, may result in plants with less resistance and problems in handling, grain quality and harvest inefficiency in the BRS Fc 104 cultivar.

Cultivar BRS FC 104, under favorable cultivation conditions such as nutrition, light, temperature and rainfall, despite its very early cycle, has an average productivity of 3.792 t ha^−1^ [42], similar to the result obtained for this work, 3.35 t ha^−1^. Cultivar BRS MG Realce showed a mean lower than that described by Melo et. al., (2014) [43]; the experimental average was 1.56 t ha^−1^, with the average commonly presented for the cultivar being 2.128 t ha^−1^. These reductions in productivity parameters may be related to the low physiological vigor of the cultivar, culminating, therefore, in the formation of plants with low performance, which consequently led to a sudden reduction in productivity.

The results obtained evidence the variations in the growth and productive parameters between the BRS MG Realce and BRS FC 104 cultivars and the need for a more in-depth understanding of FP application to bean plants in order to highlight the direct effects on CO_2_ absorption, biomass accumulation and productivity, including to evaluate FP treatments in cultivation with water deficit.

According to the available literature, FP application creates a film on the leaf surface, which reduces stomatal conductance, impacting CO_2_ entry and, therefore, plant growth and productivity. In areas with a low technological level and in conditions of water deficit as a limiting factor, the use of FP can prove to be a viable alternative to reduce water loss resulting from plant transpiration. Other notes for the use of FP would be to evaluate the concentration applied and the moment of application in more cultivars, as there is evidence of interaction with the genotype.

However, artificial shading provided ideal conditions for gas exchange, growth and biomass accumulation in plants. In addition to reducing solar radiation, shading reduced temperature and favored better photosynthetic and transpiration rates, promoting an environment conducive to the healthy development of plants. Ultimately, this study could be useful to define a practical response for farmers to improve bean yield under the specific growing conditions of the Cerrado Region, in Brazil.

## 4. Materials and Methods

### 4.1. Plant Material and Growth Conditions

Two common bean cultivars were used, BRS Fc 104 and BRS MG Realce, at the Instituto Federal Goiano—Campus Ceres, Ceres, Goiás, Brazil, at the geographic coordinates latitude—15.348569 South, longitude—49.600885 West, with approximately 574 m of altitude. Both cultivars are widely cultivated in Brazil, showing morphoagronomic differences. The BRS FC 104 cultivar has a very early growing cycle, undetermined growth and higher yield (around 3.8 kg ha^−1^) compared to the BRS MG Realce cultivar.

The experiments comprised two cycles from May 2019 to January 2020. The local climate is classified in the International System of Koppen—Geiger as Aw (Savanna-type climate), with hot rainy summers and mild dry winters [44]. Soil correction and fertilization, planting seeds and covering were carried out according to Embrapa’s recommendations [45]. The seeds were treated before planting, and at 5 days after emergence (DAE), thinning was performed, leaving one seedling per pot or 240,000 plants per hectare.

Temperature, evaporation, evapotranspiration and precipitation were monitored during the experiments (Figure 3) by a local weather station. Photosynthetically active radiation under experimental conditions in full sun exceeds 2400 µmol m^−2^ s^−1^. The maximum relative humidity varied between 60 and 99% and the minimum between 15 and 66%, with little variation between environments.

### 4.2. Experimental Design and Treatments

Two distinct experiments were conducted to evaluate the efficacy of two climate change mitigation strategies on the physiology, growth and production of *Phaseolus vulgaris* L. In order to compare shading levels with the application of a foliar protector, an open-field pot experiment was performed with a total of 48 plants of the cultivar BRS Fc 104 grown in individual 12 L pots, in which 12 plants were treated with the application of a commercial calcium (Ca) and nitrogen (N) foliar protector (Protex^®^, Santa Clara Agrociência, Ribeirão Preto, SP, Brazil) and grown under full sunlight exposure. In total, 24 plants were shaded at two levels (12 plants with 50% and 12 plants with 70% shading), and 12 plants were grown under full sunlight exposure, corresponding to the control group. The shaded conditions were performed by using plastic nets, made of high-density polyethylene, flexible, with uniformity and shading defined by sombrite^®^. The foliar protector (Protex^®^) is a liquid formulation with nanoparticle technology, recommended to supply calcium (25%, *w*/*w*) and nitrogen (1%, *w*/*w*) to plants, aiming to protect excess solar radiation, decrease water and CO_2_ loss, and minimize heat and high light stress. In this first experiment, the foliar protector (FP) was applied twice (one at the beginning of flowering—R6; 26 DAE, and the other at the beginning of pod formation—R7; 37 DAE) at a concentration of 2%, according to the recommended dose for the common bean crop, using a solution containing 2 L of the commercial product for 100 L of syrup, and applied in a volume large enough to completely cover the plants until the surface of the leaves began to run.

Pots with a capacity of 12 L were filled with surface soil, with pH in H_2_O = 5.10 (mg dm^−3^), organic matter = 5.80 (mg dm^−3^), P = 0.5 (mg dm^−3^), K = 31.00 (mg dm^−3^), Ca^2 +^ = 0.70 (cmolc dm^−3^), Mg^2+^ = 0.70 (cmolc dm^−3^), Al^2+^ = 0.10 (cmolc dm^−3^ ), H + Al = 1.80 (cmolcdm^−3^), Ca + Mg = 1.4 (cmolcdm^−3^), Ca/Mg = 1.0 (cmolcdm^−3^), CTC = 3.3 (cmolcdm ^−3^), V = 45.11(%), Clay = 60.2%, Silt = 9.8% and Sand = 30.1%.

The second experiment aimed to study the effect of the foliar protector (FP) in different cultivars of common bean. The experiment in an irrigated production field was undertaken in a total of 252 plants of each cultivar (BRS Fc 104 cv. and BRS MG Realce cv) at different phenological stages. The treatments were applied in 36 plants of each cultivar at several phenological stages as follows: in the vegetative phase (20 DAE), flowering (34 DAE), in pod formation (46 DAE), grain filling (52 DAE), and by combinations of sprays in pod formation + grain filling (46 and 52 DAE), and in the vegetative + flowering + pod formation + grain filling (20, 34, 46 and 52 DAE). An untreated control group for each cultivar was also composed of 36 plants.

Based on soil analysis and the recommendations from Souza and Lobato (2004) [45], for the bean crop, a planting adduction was carried out, providing 120 Kg ha^−1^ of P_2_O_5_ using simple superphosphate (21% of P_2_O_5_). After 12 DAE, a formulated fertilizer 20-00-20 and urea were applied following the same parameters of the sowing fertilization.

Sowing occurred on 7 November 2019, where 5 seeds were placed per pot, the seeds were treated with nematicidal insecticide (Cropstar^®^, Bayer, Leverkusen, Germany) at a dosage of 0.5 L for 100 kg of seeds. The seedlings were thinned at 5 DAP, leaving only one seedling per pot.

### 4.3. Irrigation Management

**Figure 4:**
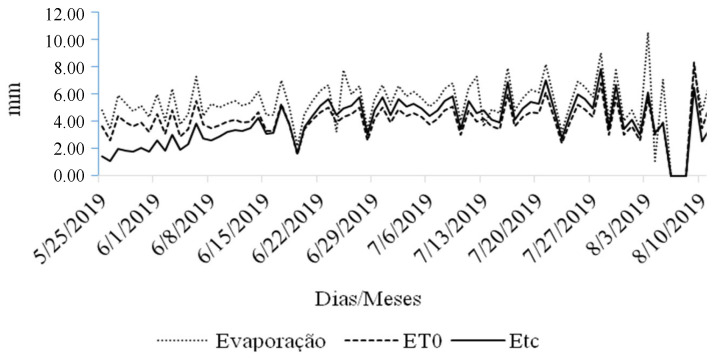
Evaporation of free water in the Class A Tank (ECA), reference evapotranspiration and evapotranspiration of common bean irrigated by center pivot. Ceres, 2019.

In the pot experiment, irrigation was supplied by drip irrigation, and in the production field, it was performed with a central pivot irrigation system, adopting Class A tank management. In the production field, from May to August, there was no precipitation rainfall and the crop’s water requirement was provided via irrigation using a center pivot system. Figure 4 shows the values of evaporation of water in the Class A tank ETc and ETo during the experiment.

### 4.4. Photosynthesis and Transpiration Measurements

Photosynthesis and transpiration measurements were performed in tree lectures in each of the four leaves of four plants 5 and 11 days after the first and second application, respectively, with an Infrared Gas Analyzer (IRGA), CID Bio-Science, Inc., model CI-340 Handheld Photosynthesis System, Camas, WA, USA, in the first fully expanded trefoil, between 09:00 and 11:00, and on sunny and cloudless days.

### 4.5. Growth and Production Traits

The evaluation of growth and harvest of pods were at the end of the plant cycle, corresponding to 65 DAE for plants grown in full sun exposure and at 68 DAE for plants grown in environments with 50% and 70% shading. The plant height, insertion height of the first pod, diameter of the stem in the middle third of the stem, number of sprouts, distance between the nodes (from the first to the second node, the second to the third node, the third to fourth node, from fourth to fifth), productivity, number of pods per plant, number of grains per plant, mass of grains per plant and dry mass of plant were evaluated.

In the production field experiment, the height of insertion of the first pod, the number of sprouts, the diameter of the stem at one cm from the ground level, the distance between the nodes, the number of pods per plant, the number of grains per plant, and productivity were evaluated.

### 4.6. Statistical Analysis

Data were submitted to analysis of variance with an F test at a 5% probability of error and the means of treatments were compared by the Tukey test at a 5% probability of error, using the Sisvar 5.6 Software [46].

## 5. Conclusions

The application of foliar protector did not prove as effective as artificial shading in mitigating the adverse effects of excessive radiation and temperature on the physiology, growth, and yield of common bean plants cv. BRS Fc 104.

In field conditions, applying foliar protector at different phenological stages did not increase the growth and productivity of the two bean cultivars (BRS Fc 104 and BRS MG Realce).

## Figures and Tables

**Table 1:** Growth and yield variables of common bean in the pot experiment (BRS Fc104) at 65 DAE for control and 68 DAE for shading.

Treatments	PH (cm)	NB	SD (mm)	FPIH (cm)	DBFSN	DBSTN (cm)	DBTFN (cm)	DBFFN (cm)	NPP	NGP	GMP (g)	PDM (g)
Control	71.25 ± 13.08 b	3.08 ± 0.63 a	4.13 ± 0.75 a	9.41 ± 2.08 a	8.18 ± 1.7 a	6.86 ± 1.3 b	6.6 ± 1.2 b	8.18 ± 1.55 b	3.2 5 ± 0.98 bc	3.8 ± 1.08 b	0.49 ± 0.15 b	8.08 ± 1.58 ab
FP	52.5 ± 11.01 b	3.17 ± 0.66 a	3.7 ± 0.67 a	10.9 ± 2.79 a	7.41 ± 1.4 a	5.7 ± 1.1 b	6.49 ± 1.3 b	9.40 ± 1.86 b	1.8 ± 0.68 c	3.75 ± 1.6 b	0.45 ± 0.24 b	5.75 ± 1.07 b
50% shading	149.92 ± 12.74 a	4.92 ± 0.5 a	5.2 ± 0.15 a	9.62 ± 1.17 a	9.28 ± 0.5 a	10.96 ± 0.58 a	13.14 ± 0.99 a	20.34 ± 1.6 a	8.2 5 ± 1.0 b	22.92 ± 3,44 b	4.26 ± 0.8 b	13.3 ± 1.88 a
70% shading	143.5 ± 10.04 a	4.5 ± 0.34 a	5.38 ± 0.14 a	11.83 ± 1.08 a	10.94 ± 0.49 a	10.38 ± 0.4 a	13.3 ± 0.89 a	20.03 ± 1.5 a	18.58 ± 2.17 a	56.58 ± 9.97 a	13.58 ± 2.95 a	13.75 ± 1.75 a

Means ± SE followed by the same letter in the columns do not differ statistically by Tukey’s test at a 5% probability of error. PH—plant height; NB—number of branches; SD—stem diameter; FPIH—First pod insertion height; DBFSN—distance between the first and the second node; DBSTN—distance between the second and the third node; DBTFN—distance between the third and the fourth node; DBFFN—distance between the fourth and the fifth node; NPP—number of pods per plant; NGP—number of grains per plant; GMP—grain mass per plant; PDM—plant dry mass.

**Table 2:** Pearson’s correlation between growth and yield variables of common bean cultivated with artificial shading and application of sunscreen.

	NB	SD	DBFSN	DBSTN	DBTFN	DBFFN	NPP	NGP	PH	FPIH	GMP	PDM
NB	1.00											
SD	0.77	1.00										
DBFSN	0.65	0.86	1.00									
DBSTN	0.68	0.84	0.79	1.00								
DBTFN	0.61	0.71	0.65	0.81	1.00							
DBFFN	0.62	0.65	0.58	0.78	0.89	1.00						
NPP	0.37	0.45	0.36	0.45	0.53	0.52	1.00					
NGP	0.26	0.31	0.22	0.36	0.46	0.50	0.92	1.00				
PH	0.64	0.73	0.56	0.78	0.72	0.69	0.63	0.51	1.00			
FPIH	0.43	0.61	0.53	0.43	0.33	0.30	0.15	0.04	0.34	1.00		
GMP	0.21	0.26	0.17	0.30	0.39	0.43	0.88	0.99	0.43	0.03	1.00	
PDM	0.62	0.68	0.55	0.66	0.56	0.55	0.36	0.19	0.78	0.39	0.12	1.00

PH—plant height; NB—number of branches; SD—stem diameter; FPIH—First pod insertion height; DBFSN—distance between the first and the second node; DBSTN—distance between the second and the third node; DBTFN—distance between the third and the fourth node; DBFFN—distance between the fourth and the fifth node; NPP—number of pods per plant; NGP—number of grains per plant; GMP—grain mass per plant; PDM—plant dry mass.

**Table 3:** Number of branches (NB), stem diameter (SD), distance between nodes (DBN), number of pods per plant (NPP), number of grains per plant (NGP) and productivity of field-grown *Phaseolus vulgaris* cultivars (BRS Fc 104 and BRS MG Realce). Ceres, GO, 2019.

Cultivar	NB	SD (mm)	DBN (mm)	NPP	NGP	Productivity (t ha^−1^)
BRS Fc 104	10.56 ± 0.27 a	4.85 ± 0.05 b	21.94 ± 0.3 a	24.18 ± 0.7 a	119.04 ± 3.64 a	3.35 ± 0.97 a
BRS MG Realce	6.84 ± 0.1 b	6.47 ± 0.08 a	20.0 ± 0.28 b	18.90 ± 0.49 b	76.34 ± 2.2 b	1.56 ± 0.56 b

Means ± SE followed by the same letter in the columns do not differ statistically by Tukey’s test at 5% probability of error.

**Table 4:** Number of branches in two cultivars *Phaseolus vulgaris* with FP application at different phenological phases.

FP Application at Different Phenological Phases	Branches	
	BRS Fc 104	BRS MG Realce
Vegetative phase	11.3 ± 0.76 ab	6.9 ± 0.25 a
Flowering (R6)	9.7 ± 0.51 b	6.4 ± 0.30 a
Pod formation (R7)	9.4 ± 0.45 b	6.9 ± 0.28 a
Grain filling	10.7 ± 0.88 ab	6.7 ± 0.26 a
Pod formation + grain filling	9.9 ± 0.57 b	7.0 ± 0.27 a
Vegetative + flowering + pod formation + grain filling	10.0 ± 0.71 b	6.6 ± 0.35 a
Control	12.7 ± 0.86 a	7.3 ± 0.36 a

Means ± SE followed by the same letter in the columns do not differ statistically by Tukey’s test at 5% probability of error.

**Table 5:** Productivity the two cultivars *Phaseolus vulgaris* with FP application at different phenological phases.

FP Application at Different Phenological Phases	Productivity (t ha^−1^)	
	BRS Fc 104	BRS MG Realce
Vegetative phase	3.25 ± 0.51	1.69 ± 0.21
Flowering (R6)	3.69 ± 0.16	1.25 ± 0.14
Pod formation (R7)	2.87 ±0.37	1.81 ± 0.32
Grain filling	3.78 ± 0.27	1.31 ± 0.08
Pod formation + grain filling	3.02 ± 0.37	1.54 ± 0.13
Vegetative + flowering + pod formation + grain filling	3.32 ± 0.54	1.68 ± 0.23
Control	3.55 ± 0.35	1.64 ± 0.14

The means ± SE did not differ statistically according to Tukey’s test at a 5% significance level.

## Data Availability

Data is contained within the article.

## References

[1] Reis A.F., Schmiele M. (2019). Características e potencialidades dos frutos do Cerrado na indústria de alimentos. Braz. J. Food Technol..

[2] Silva J.G. Cultivo do Feijão: Semeadura. https://www.embrapa.br/agencia-de-informacao-tecnologica/cultivos/feijao/producao/semeadura.

[3] Gerlach G.A., Arf O., Corsini D.C., Silva J.C., Coletti A.J. (2013). Análise econômica da produção de feijão em função de doses de nitrogênio e coberturas vegetal. Pesqui. Agropecu. Trop..

[4] Silva D.A., Pinto-Maglio C.A.F., Oliveira E.C., Reis R.L.M., Carbonell S.A.M., Chiorato A.F. (2020). Influence of high temperature on the reproductive biology of dry edible bean (*Phaseolus vulgaris* L.). Sci. Agric..

[5] Daniel E. (1997). The temperature dependence of photoinhibition in leaves of *Phaseolus vulgaris* (L.): Influence of CO_2_ and O_2_ concentrarions. Plant Sci..

[6] Ribeiro R.V., Santos M.G., Souza G.M., Machado E.C., Oliveira R.F., Angelocci L.R., Pimentel C. (2004). Environmental effects on photosynthetic capacity of bean genotypes. Pesqui. Agropecu. Bras..

[7] Bisbis M.B., Gruda N., Blanke M. (2018). Potential impacts of climate change on vegetable production and product quality—A review. J. Clean. Prod..

[8] Gharaghani A., Javarzari A.M., Vahdati K. (2018). Kaolin particle film alleviates adverse effects of light and heat stresses and improves nut and kernel quality in Persian walnut. Sci. Hortic..

[9] Dash P.K., Chase C.A., Agehara S., Zotarelli L. (2020). Heat stress mitigation effects of kaolin and s-abscisic acid during the establishment of strawberry plug transplants. Sci. Hortic..

[10] Salib N.C. (2018). Respostas Fisiológicas de Soja à Aplicação de Caulim e Carbonato de Cálcio. Master’s Thesis.

[11] Jifon J.L., Syvertsen J.P. (2003). Kaolin Particle Film Applications Can Increase Photosynthesis and Water Use Efficiency of ‘Ruby Red’ Grapefruit Leaves. J. Am. Soc. Hortic. Sci..

[12] Tommaso F., Simone S., Cecilia S., Sergio T., Alberto P., Paolo S., Eugenio M., Stefano P. (2019). Understanding kaolin effects on grapevine leaf and whole-canopy T physiology during water stress and re-watering. J. Plant Physiol..

[13] Dinis L.-T., Bernardo S., Luzio A., Pinto G., Meijón M., Pintó-Marijuan M., Cotado A., Correia C., Moutinho-Pereira J. (2018). Kaolin modulates ABA and IAA dynamics and physiology of grapevine under Mediterranean summer stress. J. Plant Physiol..

[14] Boari F., Donadio A., Pace B., Schiattone M.I., Cantore V. (2016). Kaolin improves salinity tolerance, water use efficiency and quality of tomato. Agric. Water Manag..

[15] Rosati A. (2007). Physiological Effects of Kaolin Particle Film Technology: A Review. Funct. Plant Sci. Biotechnol..

[16] Bernardo S., Dinis L.-T., Machado N., Barros A., Pitarch-Bielsa M., Gómez-Cadenas A., Moutinho-Pereira J. (2021). Kaolin impacts on hormonal balance, polyphenolic composition and oenological parameters in red grapevine berries during ripening. J. Berry Res..

[17] Salama D.M., Abd El-Aziz M.E., Shaaban E.A., Osman S.A., Abd El-Wahed M.S. (2022). The impact of nanofertilizer on agro-morphological criteria, yield, and genomic stability of common bean (*Phaseolus vulgaris* L.). Sci. Rep..

[18] Franco-Lagos C.L., Sánchez E., Palacio-Márquez A., Pérez-Álvarez S., Terrazas-Gómez M., Villalobos-Cano O., Ramírez-Estrada C.A. (2023). Efficacy of the application of boron nanofertilizer on biomass, yield, nitrogen assimilation and photosynthetic activity in green beans. Not. Bot. Horti Agrobot..

[19] Boari F., Cucci G., Donadio A., Schiattone M.I., Cantore V. (2014). Kaolin influences tomato response to salinity: Physiological aspects. Acta Agric. Scand. Sect. B—Soil Plant Sci..

[20] Kanojia A., Shrestha D.K., Dijkwel P.P. (2021). Primary metabolic processes as drivers of leaf ageing. Cell. Mol. Life Sci..

[21] Hideg É., Kálai T., Hideg K., Vass I. (1998). Photoinhibition of photosynthesis in vivo results in singlet oxygen production detection via nitroxide-induced fluorescence quenching in broad bean leaves. Biochemistry.

[22] Tsonev T., Velikova V., Lambreva M., Stefanov D. (1999). Recovery of the photosynthetic apparatus in bean plants after high-and low-temperature induced photoinhibition. Bulg. J. Plant Physiol..

[23] Casaroli D., Van Lier Q.D.J. (2015). Resposta fotossintética do feijoeiro em função da intensidade de radiação e do teor de água no solo. Rev. De Ciênc. Agroambientais.

[24] Araújo L.L.N., De Melo H.C., Castiglioni G.L., Gonçalves L.A. (2019). Intensidade de radiação influenciando características morfofisiológicas em folhas de *Tetradenia riparia* (Hochst.) Codd. Iheringia Série Bot..

[25] Schock A.A., Ramm A., Martinazzo E.G., Silva D.M., Bacarin M.A. (2014). Crescimento e fotossíntese de plantas de pinhão-manso cultivadas em diferentes condições de luminosidade. Rev. Bras. De Eng. Agríc. E Ambient..

[26] Zheng L., Van Labeke M.C. (2017). Long-Term Effects of Red- and Blue-Light Emitting Diodes on Leaf Anatomy and Photosynt Efficiency of Three Ornamental Pot Plants. Front. Plant Sci..

[27] Landi M., Zivcak M., Sytar O., Brestic M., Allakhverdiev S.I. (2020). Plasticity of photosynthetic processes and the accumulation of secondary metabolites in plants in response to monochromatic light environments: A review. Biochim. Biophys. Acta Bioenerg..

[28] Coelho D.S., Marques M.A., da Silva J.A., da Silva Garrido M., de Carvalho P.G. (2014). Respostas fisiológicas em variedades de feijão caupi submetidas a diferentes níveis de sombreamento. Braz. J. Biosci..

[29] Crawford A.J., Mclachlan D.H., Hetherington A.M., Franklin K.A. (2012). High Temperature Exposure Increases Plant Cooling Capacity. Curr. Biol..

[30] Santos P.L.D.S. (2016). Respostas Fisiológicas do Feijão-Caupi Submetidos a Restrição Hídrica e Aplicação de Óxido de Cálcio Sobre as Folhas. Master’s Thesis.

[31] Souza G.M., Balmant B.D., Vítolo H.F., Gomes K.B.P., Florentino T.M., Catuchi T.A., Vieira W.L. (2009). Estratégias de utilização de luz e estabilidade do desenvolvimento de plântulas de *Cordia superba* Cham. (Boraginaceae) crescidas em diferentes ambientes luminosos. Acta Bot. Bras..

[32] Liang X.G., Gao Z., Shen S., Matthew J., Zhang L., Zhao X., Lin S., Wu G., Chen X., Zhou S.L. (2020). Differential ear growth of two maize varieties to shading in the field environment: Effects on whole plant carbon allocation and sugar starvation response. J. Plant Physiol..

[33] Charbonnier F., Roupsard O., Le Maire G., Guillemot J., Casanoves F., Lacointe A., Vaast P., Allinne C., Audebert L., Cambou A. (2017). Increased light-use efficiency sustains net primary productivity of shaded coffee plants in agroforestry system. Plant Cell Environ..

[34] De Assis G.A., Guimarães R.J., Colombo A., Dominghetti A.W. (2014). Drip irrigation in coffee crop under different planting densities: Growth and yield in southeastern Brazil. Rev. Bras. De Eng. Agric. E Ambient..

[35] Pereira L.F., Matsumoto S.N., De Oliveira U.S., Ramos P.A.S., Teixeira E.C., Gonçalves A.N.S., Gugé R.M.A., Virgiane A.S., Vale E.S.D., Silva T.M. Manejo da supressão e estímulo à biossíntese de giberelina em cafeeiros arábica. Proceedings of the X Simpósio de Pesquisa dos Cafés do Brasil.

[36] Tibolla L.B., Schwerz F., Sgarbossa J., Elli E.F., Nardini C., Medeiros S.L.P., Schmidt D., Caron B.O. (2019). Effect of artificial shading on soybean growth and yield. Rev. Bras. De Ciênc. Agrárias.

[37] Manivannan A., Soundararajan P., Park Y.G., Wei H., Kim S.H., Jeong B.R. (2017). Blue and red light-emitting diodes improve the growth and physiology of *in vitro*-grown carnations ‘Green Beauty’ and ‘Purple Beauty’. Hortic. Environ. Biotechnol..

[38] Naznin T., Park C.H., Lefsrud M., Azad O.K. (2019). Effect of different combinations of red and blue led light on growth characteristics and pigment content of in vitro tomato plantlets. Agriculture.

[39] Oh W., Kim J., Kim Y.H., Lee I.-J., Kim K.S. (2015). Shoot Elongation and Gibberellin Contents in *Cyclamen persicum* Are Influenced by Temperature and Light Intensity. Hortic. Environ. Biotechnol..

[40] Yu Y., Stomph T.-J., Makowski D., Van Der Werf W. (2016). A meta-analysis of relative crop yields in cereal/legume mixtures suggests options for management. Field Crop. Res..

[41] Nassary E.K., Baijukya F., Ndakidemi P.A. (2020). Productivity of intercropping with maize and common bean over five cropping seasons on smallholder farms of Tanzania. Eur. J. Agron..

[42] Melo L.C., Abreu A.F.B., Del Peloso M.J., Pereira H.S., Faria L.C., Ramalho M.A.P., Carneiro J.E.S., Paula Júnior T.J., Pereira Filho I.A., Moreira J.A.A. (2017). BRS FC104: Cultivar de Feijão-Comum Carioca Superprecoce. Comun. Técnico.

[43] Melo L.C., Abreu A.F.B., Del Peloso M.J., Pereira H.S., Faria L.C., Ramalho M.A.P., Carneiro J.E.S., Paula Júnior T.J., Pereira Filho I.A., Moreira J.A.A. (2014). BRSMG Realce: Cultivar de Feijão Comum de Grãos Rajados Adaptada a Colheita Mecânica Direta. Tecnologias Para a Sustentabilidade da Cultura do Feijão. https://www.embrapa.br/busca-de-publicacoes/-/publicacao/992439/brsmg-realce-cultivar-de-feijao-comum-de-graos-rajados-adaptada-a-colheita-mecanica-direta.

[44] Cardoso M.R.D., Marcuzzo F.F.N., Barros J.R. (2014). Climatic Classification of Köppen-Geiger for the State of Goias and Federal District. Acta Geográfica.

[45] Souza D.M.G., Lobato E. (2004). Cerrado: Correção do solo e adubação. EMBRAPA Informações Tecnológicas.

[46] Ferreira D.F. (2011). Sisvar: A computer statistical analysis system. Ciênc. Agrotec..

